# Regulations of Glycolytic Activities on Macrophages Functions in Tumor and Infectious Inflammation

**DOI:** 10.3389/fcimb.2020.00287

**Published:** 2020-06-12

**Authors:** Qing Yu, Yufei Wang, Lin Dong, Ying He, Ruichen Liu, Qiuli Yang, Yejin Cao, Yuexin Wang, Anna Jia, Yujing Bi, Guangwei Liu

**Affiliations:** ^1^Key Laboratory of Cell Proliferation and Regulation Biology, Ministry of Education, Institute of Cell Biology, College of Life Sciences, Beijing Normal University, Beijing, China; ^2^State Key Laboratory of Pathogen and Biosecurity, Beijing Institute of Microbiology and Epidemiology, Beijing, China

**Keywords:** glycolytic activity, macrophages differentiation, macrophage function, cancer, bacterial infection, infectious inflammation

## Abstract

Macrophages differentiated into a classically activated (M1) or alternatively activated phenotype (M2) in infection and tumor, but the precise effects of glycolysis and oxidative phosphorylation (OXPHOS) metabolic pathway remain unclear. Herein, the effects of glycolysis or OXPHOS on macrophage polarizations were investigated using a pharmacological approach in mice. 2-Deoxy-D-glucose (2-DG) treatments, which blocks the key enzyme hexokinase of glycolysis, efficiently inhibits a specific switch to M1 lineage, decreasing the secretion of pro-inflammatory cytokines and expressions of co-stimulatory molecules associated with relieving infectious inflammation *in vitro and in vivo*. Glycolytic activation through the hypoxia-inducible factor-1α (HIF-1α) pathway was required for differentiation to the M1 phenotype, which conferred protection against infection. Dimethyl malonate (DMM) treatment, which blocks the key element succinate of OXPHOS, efficiently inhibits a specific switch to M2 lineage when macrophages receiving M2 stimulation, decreasing the secretion of anti-inflammatory cytokine and CD206 expressions. Mitochondrial dynamic alterations including mitochondrial mass, mitochondrial membrane potential (Dym) and ROS productions were critically for differentiation to the M2 phenotype, which conferred protection against anti-tumor immunity. Glycolysis is also required for macrophage M2 differentiation. Thus, these data provide a basis for a comprehensively understanding the role of glycolysis and OXPHOS in macrophage differentiation during anti-infection and anti-tumor inflammation.

## Introduction

Macrophages are professional antigen-presenting cells (APCs) that play major role in both inflammatory diseases and cancer (Murray, 2017). As sentinels of the immune system, macrophages continuously sample changes in the microenvironment, including barrier breakage, shifts in metabolism due to nutrient depletion, increase in oxygen consumption (hypoxia) (Murray, 2017). Macrophages sense their microenvironment through an array of molecular sensors through pattern recognition receptors (Jackaman et al., 2017). Sensing of altered microenvironment and cellular metabolism often results in skewing of immune function from homeostasis to inflammatory response (Jackaman et al., 2017). As a result, macrophages with different functionality and phenotype are recruited to the tissue. Recent studies have indicated that metabolic regulation is tightly linked to macrophages development, differentiation and functions (Liu et al., 2006; Zhu et al., 2014). The distinct metabolic profiles of macrophage functional differentiation are intimately linked to their status and functions. Understanding the metabolic status and the relationship with functional differentiation of macrophages would allow us to develop novel immunomodulatory therapies to inflammatory diseases and cancer.

Glucose metabolism is an important way of energy supply for macrophages in the body (Liu et al., 2006; Byles et al., 2013; Zhu et al., 2014). Glucose can be used to fuel Adenosine triphosphate (ATP) production through two linked metabolic pathways: glycolysis and oxidative phosphorylation (OXPHOS; including the tricarboxylic acid cycle [TCA]) (Liu and Yang, 2013; Su et al., 2015; Shakespear et al., 2018). Glycolysis is an almost universal way to convert glucose into pyruvate. In aerobic organisms, pyruvate enters mitochondria, where it is completely oxidized by oxygen to carbon dioxide and water. Most of its potential energy is preserved in the form of ATP (Liu and Yang, 2013; Li et al., 2019). In the absence of enough oxygen, pyruvate will be reduced to many products by NADH, especially lactic acid in animals and ethanol in yeast. Oxidative phosphorylation (OXPHOS) refers to the process of ATP synthesis driven by the energy released by the oxidation steps of organic substances including sugar, lipid, and amino acid in the decomposition process (Zhang et al., 2018) In eukaryotic cells, oxidative phosphorylation occurs in mitochondria (Liu and Yang, 2013; Zhang et al., 2018). Systems involved in OXPHOS are distributed in the inner membrane of mitochondria in the form of complexes, forming a respiratory chain, also known as electron transfer chain (Smeitink et al., 2001; Levine and Puzio-Kuter, 2010; Saha et al., 2017). Glycolysis pathway is highly upregulated in rapidly growing cancer cells, which is the first phenomenon described by Otto Warburg (Levine and Puzio-Kuter, 2010). It is usually called Warburg effect. It is a phenomenon that highly proliferative cells tend to transfer to aerobic glycolysis even when there is enough oxygen (Wang et al., 2014; Huang et al., 2016; Phan et al., 2017). In aerobic glycolysis, NADH regenerates by reducing pyruvate to lactate via lactate dehydrogenase. Enzymes in glycolysis pathway are potential targets for cancer treatment (Wang et al., 2014). Although the importance of glycolysis and OXPHOS in the energy supply of immune cells has attracted considerable attention (O'Neill et al., 2016), their precise roles in regulating the differentiation of macrophages and in regulating infection and tumorigenic inflammation remain unclear.

In this study, we used pharmacological methods to observe the effects of glycolysis and OXPHOS on the functional differentiation of macrophages *in vitro* and *in vivo*. It was found that HIF1α-dependent glycolysis is important for M1 macrophage differentiation and plays critical roles in anti-bacterial infection. In addition, OXPHOS and mitochondrial homeostasis dynamics are required for regulating M2 macrophage differentiation in anti-tumor immunity.

## Materials and Methods

### Mice and Treatments

C57BL/6 (B6) wild-type (WT) mice used in the experiments were aged 8–12 weeks and obtained from Beijing Weitonglihua Experimental Animal Center. *Hif1*α^flox/flox^ mice (on the C57BL/6 genetic background) crossed with *Lyz-cre* mice to obtain *Hif*α^−/−^ mice. All animal experiments were performed in accordance with protocols approved by the Animal Ethics Committee of College of Life Sciences, Beijing Normal University. WT mice were treated as previously described (Saha et al., 2017). Briefly, WT mice were daily injected with dimethyl malonate (DMM; 160 mg/kg/mouse), or 2-deoxy-D-glucose (2-DG; 2 g/kg/mouse) in 0.1 mL phosphate-buffered saline (PBS) into the intraperitoneal cavity. As control, mice were intraperitoneally (i.p.) injected with 0.1 mL PBS.

### Cells and *in vitro* Cell Culture

The cell lines, B16F10 and L929, were obtained from ATCC and cultured in RPMI 1640 medium (Corning incorporated, New York, USA) supplemented with 10% fetal bovine serum (FBS) and 1% penicillin-streptomycin (Thermo Fisher Scientific, Waltham, MA, USA) as described previously (Liu and Yang, 2013; Gao et al., 2017; Phan et al., 2017). For the preparation of L929 supernatant, L929 were seeded into a T75 cell culture flask (Corning incorporated, New York, USA). After incubation for 8–10 days, cells were harvested and centrifuged to acquire the supernatant.

For bone-marrow derived macrophages (BMDMs) preparation, bone marrow cells (BMs) were flushed from femur and tibia bones of the mice. After the red cells were lysed, the BMs were grown in a humidified incubator at 37°C with conditional medium for 7 days containing 64% Dulbecco's modified eagle media (DMEM), 10% FBS, 1% penicillin-streptomycin, 25% L929 supernatant. Differentiated BMDMs were subjected to flow cytometry assays to determine the purity of macrophages. The percentages of macrophages (CD11b^+^F4/80^+^ cells) were more than 90%.

### Antibodies and Reagents

Antibodies for iNOS (D6B6S) and β-Actin (8H10D10), used for immunoblotting assays, were from Cell Signaling Technology (Danvers, MA, USA). Antibodies used for flow cytometry assays are fluorescently labeled. Fluorescently labeled antibodies for mouse F4/80 (BM8), mCD11b (M1/70), mCD8 (53–6.7), mCD86 (GL1), mCD80 (16-10A1) were purchased from eBioscience (San Diego, CA, USA). Fluorescently labeled antibodies for mCD45 (TU116), mCD44 (IM7), CD62L (MEL-14) were ordered from BD Biosciences (San Diego, CA, USA). Fluorescently labeled anti-mCD4 (RM4-4) was ordered from Biolegend (San Diego, CA, USA).

Cytofix/Cytoperm Golgi Stop Kit with BS GolgiStop™ used for intracellular staining was purchased from BD Biosciences (San Diego, CA, USA). Fixation/Permeabilization Solution Kit used for Foxp3 staining was from eBioscience (San Diego, CA, USA). Fluorescently labeled anti-TNFα (MP6-XT22), anti-IFNγ (XMG1.2) anti-IL-12p40 (C8.6), Foxp3 (NRRF-30) was purchased from eBioscience (San Diego, CA, USA). Anti-CD206 (C068C2) was from Biolegend (San Diego, CA, USA). Fluorescently labeled antibodies to Glut1 (EPR3915). Primary anti-succinate dehydrogenase subunit A (SDHA; 2E3GC12FB2AE2; used for flow cytometry analysis) and goat anti-rabbit IgG H&L (Alexa Fluor® 488) was purchased from Abcam (Mountain View, CA, USA).

### SDHA Knockdown With RNA Interference

As described previously (Coppo et al., 2016; Zhang et al., 2018; Dreschers et al., 2019; Jung et al., 2019), a gene-knockdown lentiviral construct SDHA short hairpin RNA shRNA (m) Lentiviral Particles (sc-61835-V, Santa Cruz Biotechnology, Dallas, Texas, USA) were used according to the manufacturer's instructions. Sorted BMDMs were infected with recombinant lentivirus, selected stable clones expressing the shRNA via puromycin dihydrochloride (sc-108071, Santa Cruz Biotechnology, Dallas, Texas, USA) selection, the SDHA expression was confirmed using quantitative PCR. The sorted macrophages with either control or shRNA vectors were used for functional assay.

### *L. monocytogenes* Infection

Age- and sex-matched WT mice (6–10 weeks old), treated with 2-DG or not, were injected intravenously with 3 × 10^5^ colony-forming units (CFU) of *L. monocytogenes*. 48 h later, the mice were killed to analyze the phenotypes of T cells in the peripheral lymph nodes (PLNs), mesenteric lymph nodes (MLNs), and spleens. Infected spleens and livers were harvested and homogenized in PBS. After serial dilutions, homogenates were plated on LB agar plates and incubated at 37°C overnight. Meanwhile, organs targeted by *L. monocytogenes* (spleen and liver) were fixed in 4% paraformaldehyde, embedded in paraffin, made into slices and stained with Hematoxylin-eosin (H&E).

For macrophage function analysis, macrophages in peritoneal cavity and target organs (spleen and liver) were extracted as previously described (Shi et al., 2019). Briefly, spleen and liver were digested with collagenase for 30 min at 37°Cafter cut into pieces with surgical scissors. Peritoneal cells, splenocytes and hepatocytes were then subjected to intracellular staining and flow cytometry analysis.

### Mouse Tumor Models

Age- and sex-matched WT mice (6–10 weeks old), treated with DMM or not, were injected subcutaneously with 2 × 10^5^ B16F10 melanoma cells and monitored for tumor growth. Mice were killed before their tumor size reached 225 mm^2^ according to protocols approved by the Animal Ethics Committee of College of Life Science, Beijing Normal University. 14 days later after injection, all mice were killed for flow cytometry assays of T cells and macrophages from the draining lymph nodes (dLNs) and tumors, respectively. The tumors were fixed in 4% paraformaldehyde, embedded in paraffin, made into slices and stained with H&E. Meanwhile the paraffin slices were also used for immunohistochemically (IHC) staining. The antibody used in IHC staining were as follows: mouse anti-CD11b (Google Biology, Wuhan, Hubei, China), mouse anti-F4/80 (Google Biology, Wuhan, Hubei, China). IHC-toolbox within ImageJ was used to analyze with IHC image (Liu and Yang, 2013). For training samples, randomly captured 10 images under 40 X magnitude. For experiment samples, randomly selected 5 images from the middle, right-top, left-top, right-bottom, and left bottom from each slice under 40 X magnitude. The brown color area was measured, and the percentages of brown color area of each image was calculated.

### Metabolic Assays

The respiratory burst indicated by proton production rate (PPR) was measured as previously described (Liu et al., 2013a). Briefly, macrophages were sorted from peritoneal cells and incubated with dihydrorhodamine (1 M, Sigma-Aldrich-P8139, St.Louis, MO, USA). Samples were incubated at 37°C for 15 min and subjected to flow cytometry analysis. Oxygen consumption rate (OCR) was measured with an XF_e_24 extracellular flux analyzer (Seahorse Bioscience) according to the manufacturer's instructions as described previously (Lu et al., 2018). In brief, macrophages sorted from peritoneal exudate cells activated with lipopolysaccharide (LPS) for 24 h, or IL-4 for 48 h were seeded in XF_e_24 microplates (5 × 10^5^) to immobilize the cells. Wash the cells with XF base medium with glucose (10 mM), sodium pyruvate (1 mM), L-glutamine (2 mM) (referred to as the assay medium). After incubation in the assay medium in an incubator without CO_2_ for 1 h, cells were subjected to oxygen consumption assays with a Mito stress test kit (Seahorse Biosciences). oligomycin (1 μM), FCCP (1 μM), rotenone/antimycin A (0.5 μM) were added into medium subsequence. The data were acquired on the Seahorse XF-24 and analyzed on the Wave.

### Flow Cytometry Assays

For flow cytometry analysis of surface markers, cells were stained with buffer as suggested in the antibody protocols. For cell intracellular staining, Cytofix/Cytoperm Golgi Stop Kit with BS GolgiStop^TM^ and Fixation/Permeabilization Solution Kit were used according to the manufacturer's instructions. For macrophage intracellular staining, cells isolated from the indicated organs were stimulated with LPS or IL-4 to induce the polarization of macrophages. Then polarized macrophages were restimulated with LPS for 5 h, together with GolgiStop. For T cell intracellular staining, cells isolated from the indicated organs were stimulated with phorbol-12-myristate-13-acetate (PMA) and ionomycin with GolgiStop. After surface staining and cleaning, cells were fixed and infiltrated with Fixation/Permeabilization solution immediately. The cells were then stained with fluorescent labeled antibodies for 30 min and analyzed on ACEA new cells (ACEA Biosciences). Then use novoexpress to collect and analyze data. The expression of the molecule was analyzed by flow cytometry. To quantify the expression in the whole positive cell population, the mean fluorescence intensity (MFI) was analyzed. To compare the ratio of different cell populations, the percentage of positive or negative cells was quantified.

### Quantitative Real-Time PCR and Immunoblotting Assays

RNA was extracted with TRI Reagent (Sigma-Aldrich, St.Louis, MO, USA) from peritoneal exudate macrophages (PEMs). Complementary DNA (cDNA) was synthesized using the PrimeScript™ RT Master Mix (Perfect Real Time) (Takara, Kusatsu, Shiga, Japan). An ABI Q6 Flex Real-time PCR system was used for quantitative PCR with primers from Applied Biosystems. *Hif1*α gene specific primers used in this study are as follows. Forward primer: ccagcagacccagttacaga; Reverse primer: tgagtgccactgtatgctga. The individual gene expression was calculated was normalized to the expression of *Hprt*. The primers used for *Hprt* were as follows. Forward primer: agtacagccccaaaatggttaag; Reverse primer: cttaggctttgtatttggcttttc. For relative quantification, SYBR® Premix ExTaq^TM^ (Perfect RealTime) (Takara, Kusatsu, Shiga, Japan) was used. The results were analyzed with An ABI Q6 Flex Real-time PCR system.

Immunoblotting assays were performed as described previously (Liu et al., 2016). To be brief, proteins were extracted with RIPA Lysis Buffer (Beyotime Biotechnology, Shanghai, China). After boiled in 100°C for 10 min, denatured proteins were subjected to dodecyl sulfate sodium salt-polyacrylamide gel electrophoresis (SDS-PAGE). Proteins were transferred from the gel to polyvinylidene fluoride (PVDF) film. After incubation with 5% bovine serum albumin (BSA), protein-specific primary antibodies and horseradish peroxidase (HRP)-conjugated goat anti-rabbit or goat anti-mouse IgG H&L, protein expression levels were detected with chemiluminescence apparatus.

### Fluorescence Microscopy

For live-cell imaging, BMDMs (5 × 10^5^), pretreated with or without DMM, were seeded on 35 mm glass-bottom dish (NEST, Wuxi, Jiangsu, China) and stimulated with IL-4 for 48 hrs. Then cells were co-cultured with mitoTracker Greeen (50 nM) and TMRM (100 nM) at 37°C for 30 min. Hochest was used to stain the nuclei. After the cells were washed with warmed PBS for 3 times, microscopy was performed with a LSM880 (Carl Zeiss) confocal microscope. The data were collected using Carl Zeiss software ZEN 2010 (black edition), and the mitochondrial lengths were measured using Carl Zeiss software ZEN 2012 (blue edition).

### Statistical Analyses

All data are presented as the means ± SD. One-way or two-way ANOVA was used for comparisons among multiple groups with the SPSS software according to the type of data. Student's unpaired *t* test for comparison of means was used to compare two groups. *P* < 0.05 was considered to be statistically significant.

## Results

### Glycolytic Metabolism Signal Shifts During Macrophage Functional Differentiation

**Figure 1:**
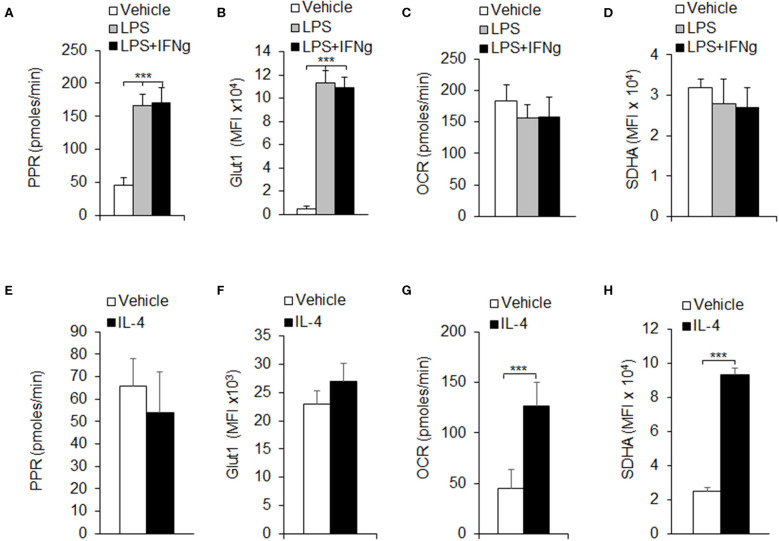
Macrophage polarizations are associated with glycolysis and OXPHOS. Peritoneal macrophages (PEMs) sorted from C57BL/6 mice were stimulated with LPS (100 ng/mL), or LPS (100 ng/mL) and IFNγ (100 ng/mL) for 24 h **(A–D)**, or IL-4 for 48 h **(E–H)**. The respiratory burst was determined by measuring the PPR **(A,E)**. The protein expression levels of Glut1 was measured by flow cytometry assays **(B,F)**. Cellular OXPHOS activity was measured by monitoring the OCR of cells **(C,G)**. The expression of SDHA, a component of ETC was determined by flow cytometry assays **(D,H)**. Representative results are based on one of three independent experiments performed with similar results. The data are presented as the mean ± SD (*n* = 3–5 mice per group). Statistical significance was measured by one-way ANOVA for comparisons among multiple groups and Student's unpaired *t* test for comparisons between two groups. ***P < 0.001, compared with the indicated groups.

Macrophages are one of the most important members in the innate immune system. Once activated, macrophages quickly proliferate and polarize into different subsets, mainly classically activated macrophages (M1) and alternatively activated macrophages (M2), to execute specific functions, which need a huge demand for energy (Liu et al., 2013b). Glycolysis and OXPHOS are important metabolic pathways for cells to produce ATP in immune responses. Our previous results and other studies have shown that pro-inflammatory signal suppresses macrophage proliferation and shifts macrophage metabolism from Myc to HIF1α dependent metabolism (Liu et al., 2016; Mills et al., 2016). To investigate the immune role of glycolysis or OXPHOS in macrophage-mediated immune responses, we purified CD11b^+^F4/80^+^ macrophages from mouse peritoneal cells and evaluated the macrophage metabolic pathway activity alterations using different macrophage polarization conditions. Here we used LPS and LPS+IFNγ which are classically considered to induce M1 macrophage polarization and IL-4 cytokines that are considered to induce M2 macrophage polarization (Liu et al., 2015). The glycolytic pathway activity of polarized macrophages was measured by the PPR, and the OXPHOS pathway activity was measured by the OCR. Results showed that LPS or LPS+IFNγ stimulation significantly increased the PPR (Figure 1A). Meanwhile, the protein levels of glucose transporter 1 (Glut1), an important member in glycolysis, were notably up-regulated on LPS or LPS+IFNγ stimulation (Figure 1B). However, neither the OCR, nor the protein levels of SDHA, an important component in electron transfer chain (ETC), were changed significantly (Figures 1C,D). These results suggested that glycolytic activity was elevated during M1 macrophage polarization, but not OXPHOS. Upon IL-4 stimulation, the PPR and the protein levels of Glut1 kept unchanged (Figures 1E,F), while the OCR and the protein levels of SDHA were increased remarkably (Figures 1G,H), indicating that OXPHOS activity was elevated during M2 macrophage polarization, but not glycolysis. Thus, the differentiation of different subtypes of macrophages may be related to the regulation of glycolysis or OXPHOS pathway.

### Glycolysis Is Required for M1 Macrophage Differentiation

**Figure 2:**
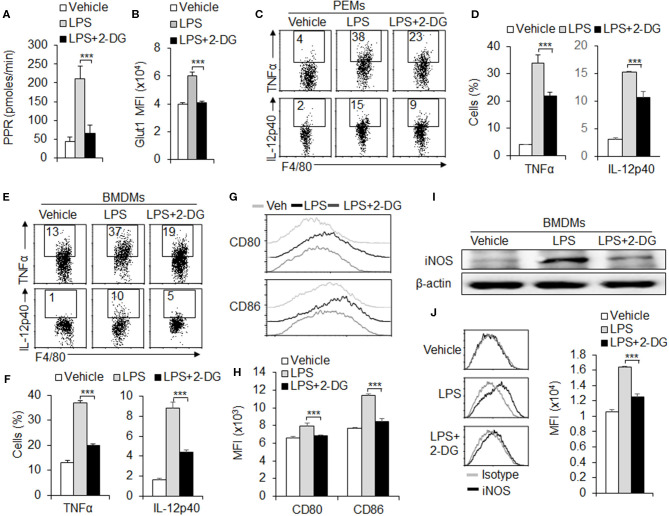
Glycolysis is required for M1 macrophage polarization. Peritoneal macrophages (PEMs) sorted from C57BL/6 mice were stimulated with LPS (100 ng/mL) for 24 h, with or without glycolysis inhibitor, 2-DG (1 mM). The respiratory burst was determined by measuring the PPR and the protein expression of Glut1 **(A,B)**. Pro-inflammatory cytokines TNFα and IL-12p40 were determined by flow cytometry assays **(C–D)**. Cells from bone marrow (BMs) were induced with L929 supernatant for 7 days. After stimulation with LPS for 24 h, the expressions of pro-inflammatory cytokines TNFα and IL-12p40 and co-stimulator molecules CD80 and CD86 were determined with flow cytometry **(E–H)**. iNOS expressions were analyzed with Immunoblotting and flow cytometry **(I,J)**. Representative results are based on one of three independent experiments performed with similar results. The data are presented as the mean ± SD (*n* = 3–5 mice per group). Statistical significance was measured by one-way ANOVA for comparisons among multiple groups. ***P < 0.001, compared with the indicated groups.

To further investigate the role of glycolysis in macrophage polarization, we treated the peritoneal macrophages with glycolysis inhibitor 2-deoxy-D- glucose (2-DG), and assessed the M1 macrophage subset polarization and function alteration *in vitro*. Results showed that blocking HK1/2 expressions with 2-DG treatment significantly downregulating the PPR level and the protein levels of Glut1, a molecule of glycolysis signaling pathway during M1 differentiation (Figures 2A,B and Figures S1A,B). Next, we examined the M1 macrophage function alteration with 2-DG treatments. Data from flow cytometry assays showed that 2-DG treatment significantly inhibited the production of pro-inflammatory cytokines TNFα and IL-12p40 in macrophages of peritoneal exudates (Figures 2C,D). Consistently, the BMDMs were induced with L929 supernatant for 7 days. 2-DG treatment significantly blocks the productions of pro-inflammatory cytokines TNFα and IL-12p40 (Figures 2E,F). Moreover, 2-DG treatment significantly suppressed the expressions of CD80 and CD86, con-stimulatory molecules of macrophages (Figures 2G,H). Also, 2-DG treatment significantly blocks the protein levels of inducible nitric oxide syntheses (iNOS), a typical marker of M1 macrophages differentiation (Figures 2I,J). However, 2-DG treatment did not affect the levels of OXPHOS and SDHA and the M2 marker CD206 and IL-10 expressions (Figures S2A–D). These data suggest glycolysis is required for M1 macrophages functional differentiation.

### OXPHOS Is Required for M2 Macrophage Functional Differentiation

**Figure 3:**
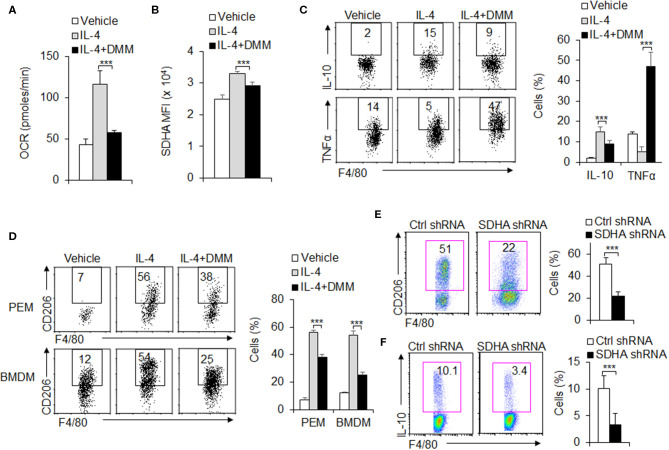
OXPHOS is required for M2 macrophage polarization. Peritoneal macrophages (PEMs) sorted from WT mice were pretreated with SDHA inhibitor DMM (160 mg/kg/mouse) and stimulated with IL-4 (10 ng/mL) for 48 h. Cellular OXPHOS activity was measured by monitoring the OCR of cells **(A)**. The protein levels of SDHA were determined by flow cytometry **(B)**. Cells from BMs from WT mice were induced with L929 supernatant for 7 days. BMDMs were then stimulated with IL-4 for 48 h and cytokines IL-10 and TNFα were determined by flow cytometry **(C)**. The CD206 expression in PEMs and BMDMs were determined by flow cytometry **(D)**. Expression of CD206 and IL-10 expression in the sorted BMDMs expressing control shRNA or SDHA shRNA vector **(E–F)**. Representative results are based on one of three independent experiments performed with similar results. The data are presented as the mean ± SD (*n* = 3–5 mice per group). Statistical significance was measured by one-way ANOVA for comparisons among multiple groups and Student's unpaired *t* test for comparisons between two groups. ***P < 0.001, compared with the indicated groups.

During the M2 macrophages differentiation, OXPHOS and SDHA signal molecule expressions are significantly upregulated and glycolysis remains unchanged compared with control groups (Figures 1E–H and Figures 3A,B). These suggest OXPHOS and SDHA signal pathway are significantly involved during the M2 macrophage functional differentiation. To further test the role of SDHA and OXPHOS signal pathway in M2 macrophage functional differentiation, PEMs are treated with SDHA inhibitor 2-dimethylimidazole (DMM), OCR levels and SDHA expressions are significantly downregulated (Figures 3A,B). Consistently, treatment with DMM caused lower anti-inflammatory cytokine IL-10 and higher proinflammatory TNFα (Figure 3C). Also, it significantly decreased the percentage and MFI of CD206, a typical M2 macrophage marker, compared with control groups in PEMs and/or BMDMs (Figure 3D and Figure S3). However, DMM treatment did not affect the levels of PPR and Glut1 and the M1 marker iNOS expressions (Figures S4A–C). Importantly, the above results with SDHA inhibitors were confirmed using SDHA shRNA (Figures 3E,F and Figure S5). Therefore, these data suggest that SDHA and OXPHOS signal pathway are critical for the M2 macrophage functional differentiation.

### OXPHOS Signal Pathway Is Critical for Maintaining the Mitochondrial Homeostasis During M2 Macrophage Differentiation

**Figure 4:**
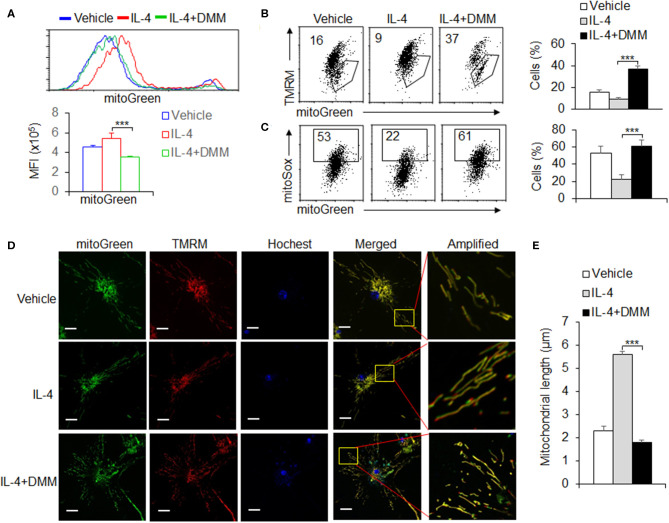
OXPHOS signal pathway is critical for maintaining the mitochondrial homeostasis during M2 macrophage differentiation. Peritoneal macrophages (PEMs) sorted from WT mice were pretreated with SDHA inhibitor DMM (10 mM) and stimulated with IL-4 for 48 hrs. The mitochondrial masses were analyzed with flow cytometry **(A)**. BMs from WT mice were induced with L929 supernatant for 7 days. BMDMs were then stimulated with IL-4 for 48 h and the percentages of mitochondrial membrane potential changes **(B)** and mitoROS production **(C)** were determined by flow cytometry. The mitochondrial morphological changes were observed by laser confocal microscope as described in Methods **(D,E)**. Representative results are based on one of three independent experiments performed with similar results. The data are presented as the mean ± SD (*n* = 3–5 mice per group). Statistical significance was measured by one-way ANOVA for comparisons among multiple groups. ***P < 0.001, compared with the indicated groups.

Mitochondrial morphologies and functions are critically involved in regulating the OXPHOS activity and cellular energy in determining the fate of cells (Martinez et al., 2013; Mills et al., 2016). We further evaluated the alterations of mitochondrial dynamics in OXPHOS signal regulation during M2 macrophages differentiation. We first stained cells with MitoTracker Green for total mitochondrial content, regardless of mitochondrial membrane potential (Dym), and found that DMM treatment recovered the increased mitochondrial mass after IL-4 stimulation, compared with control macrophages (Figure 4A). This suggests mitochondrial mass probably related with the level of mitochondrial OXPHOS. It has been known that mitochondrial mass increase could be due to accumulation of functional with Dym or dysfunctional mitochondria with loss of Dym (Liu et al., 2016). To differentiate this, we used a combination of mitoTracker Green with TMRM (Dym stain) to distinguish between respiring mitochondria and dysfunctional mitochondria and found that the increase in functional mitochondria (mitoTracker Green^high^ TMRM^high^) and the decrease in dysfunctional mitochondria (mitoTracker Green^high^ TMRM^low^) in M2 macrophages, which could be reversed by blocking SDHA and OXPHOS signaling pathway with DMM treatment. These data suggest that increased mitochondrial mass of M2 macrophages through the SDHA and OXPHOS pathways is due to the accumulation of functional mitochondria, not to the alternation of dysfunctional mitochondria. It is reported that the Dym is associated with mitochondrial ROS production (Liu and Yang, 2013; Ryan and O'Neill, 2020). To assess ROS levels in the mitochondria, we used the mitochondria-specific ROS indicator MitoSOX to selectively detect superoxide in the mitochondria of live cells. We found that MitoSOX fluorescence was downregulated in IL-4-stimulated M2 macrophages and correlated with total mitochondrial mass as indicated by MitoTracker Green staining. Blocking OXPHOS signaling pathway almost completely reversed these effects (Figure 4C), and these findings suggest that OXPHOS signal pathway is critically involved in the accumulation of functional mitochondria with low ROS production during M2 macrophages differentiation. The accumulation of low ROS-producing functional mitochondria in M2 macrophages was also visualized by live-cell imaging using both fluorescent dyes (Figure 4D). The mitochondrial fusion dynamic alteration, and formed elongated tubules, which occupied more cytoplasmic area in M2 macrophages after IL-4 stimulation, could be significantly reversed by DMM treatment (Figures 4D,E). Altogether, these data suggest OXPHOS signal pathway is critical for maintaining the mitochondrial dynamic and homeostasis during M2 macrophage differentiation.

### HIF1α-Dependent Glycolysis Is Associated With M1 and M2 Macrophage Polarization

HIF1α is reported to be a key regulator in macrophage glycolytic pathway activity. Our previous studies and other studies have also demonstrated the importance of HIF1α in macrophage polarization and metabolism (Li et al., 2018; Eddie Ip et al., 2017; Gao et al., 2017; Peruzzotti-Jametti and Pluchino, 2018; Song et al., 2019). Here we further explored the role of HIF1α in macrophage polarization and glycolytic pathway activity. We purified the macrophages from mouse peritoneal cells and stimulated the cells with LPS or LPS and IFNγ for 12 hrs. mRNA levels of *Hif1*α is determined by quantitative real-time PCR. Results showed that *Hif1*α mRNA levels were elevated on LPS or LPS and IFNγ (Figure S6A), indicating that *Hif1*α may be involved in the functional differentiation of M1 macrophages.

**Figure 5:**
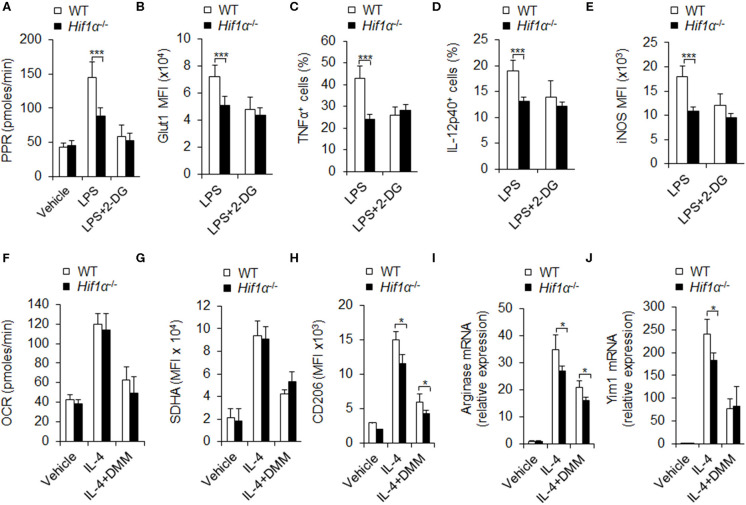
HIF1α-dependent glycolysis is associated with M1 and M2 macrophage polarization. Peritoneal macrophages (PEMs) sorted from WT mice pretreated with glycolysis inhibitor 2-DG (1 mM) or DMM (10 mM) for 1 hr were stimulated with LPS (100 ng/mL) for 12 h or IL-4 for 48 h. Cell glycolysis were measured by extracellular acidification rate **(A)**. The protein expression of Glut 1 **(B)**, intracellular staining of TNFα and IL-12p40 **(C,D)** and iNOS expression **(E)** were determined by flow cytometry. Cellular OXPHOS activity was measured by monitoring the OCR of cells **(F)**. The expression of SDHA **(G)** and CD206 **(H)** were determined by flow cytometry. mRNA expression of Arginase I **(I)** and Yim1 **(J)** were determined by qPCR. Representative results are based on one of three independent experiments performed with similar results. The data are presented as the mean ± SD (*n* = 3–5 mice per group). Student's unpaired *t* test for comparisons between two groups. *P < 0.05 and ***P < 0.001, compared with the indicated groups.

Next, we acquired PEMs from WT and *Hif1*α^−/−^ mice and determined the glycolytic activity of the cells on LPS or LPS and IFNγ stimulation. Results showed that PEMs from *Hif1*α^−/−^ mice had much lower glycolytic activity, as indicated by lower PPR and glut 1 expression (Figure S6B and Figures 5A,B). Consistently, *Hif1*α^−/−^ mice showed lower proinflammatory cytokine secretion and iNOS expression (Figures 5C–E). These data suggest HIF1α is required for macrophage glycolysis activity and M1 differentiation. While, 2-DG treatment did not alter the level of *Hif1*α mRNA (Figure S6C) and showed comparable alteration of macrophages glycolytic activities and macrophages M1 proinflammatory cytokine secretion and iNOS expression in *Hif1*α^−/−^ mice and in WT mice (Figures 5A–E). These data suggest that blocking glycolysis does not affect the expression of HIF1α and HIF1α is upstream target of glycolysis-dependent M1 macrophage functional differentiation. It is reported that mTORC1 regulates HIF1α signaling in many cells and which is essential for M1 macrophages differentiation (Eddie Ip et al., 2017). Although LPS stimulation significantly upregulated the level of p-S6, an important downstream target of mTORC1. However, 2-DG treatment did not significantly affect the expression of p-S6 (Figure S7). It is suggested that mTORC1 may regulate M1 macrophage differentiation in the upstream of glycolytic signaling pathway.

Is HIF1α related to M2 differentiation and oxidative phosphorylation? Although M2 polarization condition IL-4 treatment significantly increased oxidative phosphorylation and SDHA levels (Figures 5F,G), and also promoted M2 macrophages marker CD206, Arginase 1 and Yim1 expressions (Figures 5H–J), HIF1α deficiency significantly reduced the glycolysis PPR and glut1 expression, but not the levels of OXPHOS and SDHA, and also inhibited the expression of M2 differentiation markers CD206, Arg1, and Yim1 (Figures 5F–J and Figures S8A,B). These data suggest that HIF1α and glycolysis signaling are required for M2 macrophages differentiation. Consistently, blocking SDHA and OXPHOS activities with DMM treatment significantly inhibited OXPHOS and SDHA without affecting PPR and Glut1 levels and the level of *Hif1*α mRNA (Figures 5F,G and Figure S8C) and significantly inhibited the MFI level of macrophages M2 marker CD206 and expressions of Arg1 and Yim1 mRNA in *Hif1*α^−/−^ mice and in WT mice (Figures 5H–J). Altogether, these data collectively suggest that HIF1α-glycolysis and OXPHOS are required for M2 macrophages differentiation.

### Inhibition of Glycolysis Aggravates *L. monocytogenes* Infections and Suppresses the M1 Macrophages Differentiation

**Figure 6:**
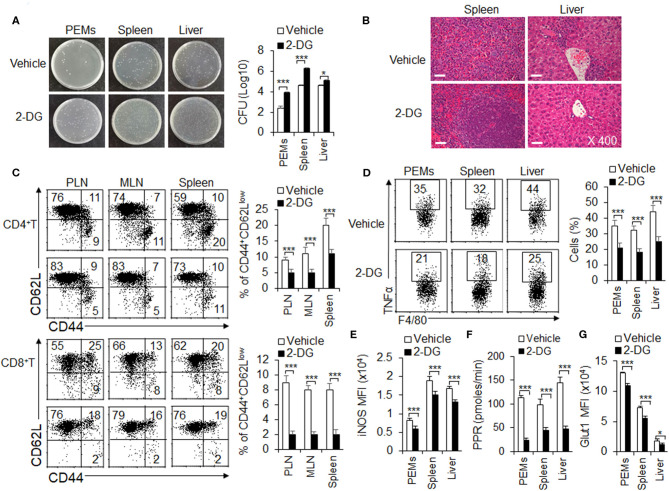
Blocking glycolysis aggravates *L. monocytogenes* infections and suppresses the M1 macrophages differentiation. WT mice were injected i.p. with 2-DG (2 g/kg weight). 4 h later, mice were i.p. injected with 3 × 10^5^ CFU of *L. monocytogenes* bacteria. 48 h after injection, mice were killed, and the CFU of mouse livers, spleens and peritoneal cavity were determined **(A)**. The H&E staining of pathological livers and spleens were shown **(B)**. The percentages of effector T cells from PLN, MLN, and spleen were determined **(C)**. Macrophages from peritoneal cavity, spleen and livers were re-stimulated with LPS (100 ng/mL) for 5 h, together with GolgiStop, and the protein expressions of pro-inflammatory cytokines TNFα and iNOS were determined by flow cytometry **(D,E)** and cell glycolysis were measured by extracellular acidification rate **(F)**, the protein expression of Glut1 was determined by flow cytometry **(G)**. Representative results are based on one of three or four independent experiments performed with similar results. The data are presented as the mean ± SD (*n* = 3–5 mice per group). Student's unpaired *t* test for comparisons between two groups. *P < 0.05 and ***P < 0.001 compared with the indicated groups.

Generally, M1 macrophages are considered to be involved in pro-inflammatory immune responses, including bacterial infection. Mouse *L. monocytogenes* infections is a classical model for evaluating the function of M1 macrophages (Gao et al., 2017). Consistent with previous studies (Miller et al., 1998), 2-DG treatment could not show direct toxic effects to *L. monocytogenes* bacterial replication when these bacterial were cultured in the presence or absence of glycolysis inhibitor 2-DG *in vitro* (Figure S9). To further investigate the significance of HIF1α-dependent glycolysis in M1 macrophage polarization and function, we challenged the WT mice, pretreated with or without glycolysis inhibitor 2-DG, with *L. monocytogenes in vivo* for 48 h (Liu et al., 2016; Li et al., 2018). At 48 h, the severity of infection was evaluated by measuring the bacterial burdens in spleen, liver and peritoneal cavity and H&E staining. As showed in the figures, mice pretreated with 2-DG displayed a markedly more severe course of infection after the challenge. 2-DG treatment significantly increased the survival of *L. monocytogenes* bacterial in PEMs, spleen and livers (Figure 6A). Microscopic and histological observations revealed a more severe pathological inflammation in spleens and livers from mice pretreated with 2-DG (Figure 6B). These results were in consistence with the T cell responses. Through flow cytometry analysis of effector T cell in peripheral lymph nodes (PLN), mesenteric lymph nodes (MLN) and spleens, the percentages of CD44^+^CD62L^low^CD4^+^T cells and CD44^+^CD62L^low^CD8^+^T cells were markedly decreased in organs from mice pretreated with 2-DG (Figure 6C). Additionally, peritoneal macrophages, splenic and hepatic macrophages from mice pretreated with 2-DG showed decreased TNFα and IL-12p40 production compared with control group (Figure 6D and Figure S10). Consistently, 2-DG treatment significantly decreased the levels of PPR, Glut1 and iNOS and suggested that blocking glycolysis treatment with 2-DG significantly inhibited the M1 macrophages differentiation (Figures 6E–G and Figure S11). Although DMM treatment significantly reduce the expression of SDHA and OXPHOS activity, but not the level of PPR and glut1, it has no significant effects on survival of *L. monocytogenes* bacterial in PEMs, spleen and livers, TNFα production and iNOS expressions (Figures S12A–F). Altogether, these data indicate that glycolysis, but not OXPHOS is required for M1 macrophage polarization in ameliorating the *L. monocytogenes* infections.

### OXPHOS Signal Pathway Promotes Tumor-Associated M2 Macrophage Differentiation in Anti-tumor Immunity

**Figure 7:**
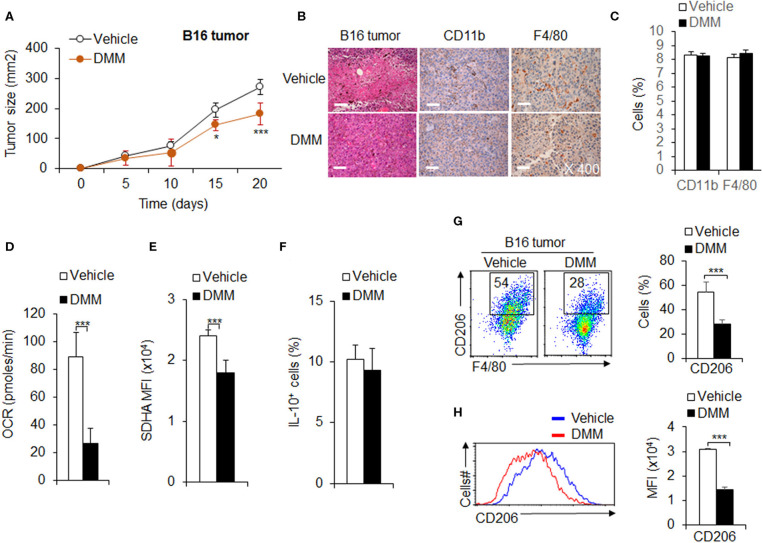
OXPHOS signal pathway promotes tumor-associated M2 macrophage differentiation in anti-tumor immunity. WT mice were intraperitoneally (i.p.) injected with DMM (160 mg/kg/mouse) for 4 h and s.c. injected with 2 × 10^5^ B16F10 melanoma cells. Tumor growth curve **(A)** and the H&E and immunohistochemistry staining of melanoma tumor was shown and positive cells percent summarized **(B,C)**. Macrophages isolated from tumor and the cellular OXPHOS activity was measured by monitoring the OCR of cells **(D)**. The expression of SDHA **(E)**, IL-10 **(F)**, and CD206 **(G,H)** in macrophages were determined by flow cytometry. Representative results are based on one of three or four independent experiments performed with similar results. The data are presented as the mean ± SD (*n* = 3–10 mice per group). Student's unpaired *t* test for comparisons between two groups. *P < 0.05 and ***P < 0.001, compared with the indicated groups.

Tumor associated M2 macrophages are reported to mediate anti-tumor immunity (Wang et al., 2019). Given the importance of OXPHOS in tumor-associated M2 macrophage polarization and function, we hypothesized OXPHOS plays an important role in M2 mediated anti-tumor immunity. We first employed a mouse model of melanoma to examine the role of OXPHOS in M2 macrophage mediated anti-tumor immunity. We injected i.p. DMM for 4 h and inoculated subcutaneous (s.c.) WT mice with B16F10 melanoma cells. DMM treatment exhibited a markedly delayed tumor growth (Figure 7A). At 14 days post-inoculation, mice were killed for flow cytometry assays of T cells and macrophages from the draining lymph nodes (dLNs) and tumors, respectively. In addition, the tumors were preserved in 4% paraformaldehyde for further pathology analysis. The IHC staining photos showed that DMM pretreated mice had comparable numbers of infiltrated immune cells, especially macrophages (Figures 7B,C). The percentage of CD44^+^CD62L^low^T cells and Foxp3^+^T cells (Figure S13) is comparable between in the dLNs of tumor-bearing mice treated by DMM or not. However, the lower production of pro-inflammatory cytokine TNFα and IFNγ in CD4^+^T cells or CD8^+^T cells in the tumor-bearing mice were significantly reversed by DMM treatment (Figure S14). These data suggest that mice pretreated with DMM suffered an alleviated inflammation in tumor, although there was no significant change in inflammatory macrophage infiltrating ratio. Consistently, blocking SDHA expression and oxidative phosphorylation activities of macrophages with DMM treatment significantly downregulated the CD206 expressions and MFI of macrophages, but not IL-10 expression in tumor-bearing mice (Figures 7D–H). DMM treatment did not affect the expression of PPR and Glut1 or change the expressions of M1 marker iNOS in macrophages (Figure S15). Blocking glycolysis activities with 2-DG treatment did not significantly inhibit tumor growth, but downregulated the macrophage M1 marker iNOS and M2 marker CD206 expressions, although not IL-10 (Figure S16). Altogether, these data suggest OXPHOS signal pathways are required for tumor-associated M2 macrophage differentiation in anti-tumor immunity.

## Discussion

Macrophages can closely coordinate their metabolic processes with their functional characteristics, enable them to develop and differentiate, and respond appropriately to a variety of pathophysiological signals in the process of infection and tumor inflammation (Rayamajhi et al., 2010; Saha et al., 2017). Inflammation is usually triggered by receptors of the innate immune system, such as toll like receptors. The initial recognition of infection was mainly mediated by macrophages in tissues, which led to the production of inflammatory mediators (Conlan, 1999). Recent studies on the metabolism of macrophage cells have shown that the metabolic spectrum has undergone profound changes in the course of macrophage activation (Kelly and O'Neill, 2015; Yeung et al., 2015; O'Neill and Pearce, 2016). For example, LPS activated macrophages will undergo glycolysis metabolism changes, while IL-4 activated macrophages will produce oxygen and phosphorus, both of which indicate that metabolic adaptation during macrophage activation is a key component of macrophage polarization, which helps them play a role in infection and tumor inflammation, but their precise roles in regulating the differentiation of macrophages and in regulating infection and tumorigenic inflammation remain unclear. In this study, we found that HIF1α-dependent glycolysis is important for M1 macrophage differentiation and plays a critical role in anti-bacterial infection. In addition, OXPHOS and mitochondrial homeostasis dynamics play an important role in regulating M2 macrophage differentiation in anti-tumor immunity (Figure S17).

Cancer cells exhibit glycolysis even in the presence of oxygen, a phenomenon known as Warburg effect. Instead, a noncancerous cell changes its metabolism from glucose oxidation to glycolysis based on its ambient oxygen concentration (Yeung et al., 2015; Jackaman et al., 2017; Murray, 2017). In this study, we investigated the glucose metabolism of primary macrophages and demonstrated that HIF-1 α-dependent glycolysis is necessary for the ATP supply of macrophages. In addition, we also found that glycolysis reprogramming plays a key role in macrophage function. Warburg effect refers to the glycolysis of cancer cells even in the presence of oxygen. On the contrary, glucose metabolism of non-cancer cells changes flexibly according to the effectiveness of molecular oxygen (Murray, 2017). A phenomenon called the Pasteur effect. The mitochondrial electron transport chain is the key to glucose oxidation, which leads to the transformation of metabolism to glycolysis under the condition of severe hypoxia (Wang et al., 2019). Previously, we have shown that HIF-1α mediated glycolysis activation is beneficial to the synthesis of ATP under severe hypoxia to guide the differentiation of MDSC in tumors (Buck et al., 2016; Wang et al., 2019). Here, we report that HIF-1 α-glycolysis axis actively changes glucose metabolism from glucose oxidation to glycolysis. This change in glycolysis can be called active glycolysis because it alters metabolic flow and has nothing to do with mitochondrial activity compared to classical glycolysis in severe hypoxia. By changing the distribution of ATP in cells, the glycolytic activity of non-cancer cells may play an important role in cell-type specific function. In this study, we found that glycolysis reprogramming under hypoxia plays an important role in macrophage mobilization, which is consistent with previous reports (Wang et al., 2019). In addition, active glycolysis may help to prevent excessive ROS production when mitochondrial respiration is impaired.

Mitochondria are dynamic organelles that often divide and fuse. Previous evidence suggests that mitochondria not only maintain the homeostasis of immune cells, but also are necessary to initiate immune responses (Palsson-McDermott et al., 2015; Weinberg et al., 2015; Buck et al., 2016; Escoll et al., 2017). Changes in mitochondrial metabolism after macrophage inflammatory therapy are essential for proper immune response (Christofk et al., 2008; Li et al., 2018). Studies as early as the 1970s have shown that inflammatory stimulation attenuates macrophage respiration by inhibiting the respiration of Compounds II and III and slowing down state III. Inflammatory stimulation also alters Krebs circulation, effectively breaking it after citrate and succinate (Colegio et al., 2014; Palsson-McDermott et al., 2015; Saha et al., 2017; Li et al., 2018). In addition, previous studies have shown that inflammatory stimulation increases mitochondrial ROS production and is an important response of macrophages to kill bacteria (Garaude et al., 2016; Peruzzotti-Jametti and Pluchino, 2018), but the mechanism was obscure. Moreover, how mitochondrial dynamics determine the subtype of immune response is still lack of research. In this study, we elucidated the important role of mitochondrial morphology in promoting the differentiation of M2 macrophages. Our study shows that mitochondrial dynamic alteration is central to determining the level of OXPHOS and the functional phenotype of macrophages. OXPHOS or succinate-dependent OXPHOS is critically involved in determining the tumor-associated M2 macrophages differentiation and takes effects in anti-tumor immunity. Studies have shown that cancer cells rely on glycolysis and are sensitive to 2-DG therapy (Levine and Puzio-Kuter, 2010; Colegio et al., 2014; Garaude et al., 2016). Although our results showed that 2-DG treatment inhibited the expression of some tumor associated M2 macrophages markers, it still did not significantly inhibit tumor growth in mice. Consistent with previous study (Zhao et al., 2017; Peruzzotti-Jametti and Pluchino, 2018), glycolysis is also required for the M2 macrophages differentiation. Moreover, HIF1α-glycolysis is also critical for the M2 macrophages differentiation. However, in our data, although blocking glycolysis with 2-DG treatment inhibited M2 macrophage differentiation, it did not show significant anti-tumor effects in tumor bearing mice. These data suggest that the regulation of glucose activity, especially OXPHOS, is very crucial in regulating the differentiation of M2 macrophages in anti-tumor immunity.

In conclusion, our results show that HIF1α-dependent glycolysis, OXPHOS and mitochondrial dynamics significantly induce the different processes of macrophage functional differentiation. Therefore, when inflammation occurs, macrophages with different functions gather in different inflammatory environments to induce immune response and eliminate inflammatory response. This study used *in viv*o and *in vitro* experimental system to clarify the changes of macrophage function guided by glucose metabolism of macrophages and to play a critical regulatory role in anti-infection and anti-cancer immunity. Therefore, these data collectively indicated that metabolic regulation is tightly linked to macrophages functional differentiation. The distinct metabolic profiles of macrophage functional differentiation are intimately linked to their status and functions in infection and cancer.

## Data Availability Statement

All datasets generated for this study are included in the article/Supplementary Material.

## Ethics Statement

All animal experiments were performed in accordance with protocols approved by the Animal Ethics Committee of College of Life Science, Beijing Normal University.

## Author Contributions

QYu, YW, LD, YH, and YB designed and conducted the experiment with cells and mice, analyzed data. RL conducted the experiments with mice and analyzed data. QYa, YC, YW, and AJ participated in discussions. QYu and YB contributed to writing the manuscript and participated in discussions. GL developed the concept, designed and conducted the experiments with cells and mice, analysed data, wrote the manuscript, and provided overall direction. All authors contributed to the article and approved the submitted version.

## Conflict of Interest

The authors declare that the research was conducted in the absence of any commercial or financial relationships that could be construed as a potential conflict of interest.
