# Supplementary material for: Regulations of Glycolytic Activities on Macrophages Functions in Tumor and Infectious Inflammation
Source: Front Cell Infect Microbiol. 2020 Jun 12;10:287. doi: 10.3389/fcimb.2020.00287 (PMC7303283; doi:10.3389/fcimb.2020.00287)
Supplement: Supplementary file 1 [file Data_Sheet_1.pdf]

Supplemental Figure

Regulations of glycolytic activities on macrophages functions in tumor and infectious inflammation

Qing Yu[1,#], Yufei Wang[1,#], Lin Dong[1,#], Ying He[1,#], Ruichen Liu[1,#], Qiuli Yang[1], Yejin Cao[1], Yuexin Wang[1], Anna Jia[1], Yujing Bi[2,*], Guangwei Liu[1,*]

[1]Key Laboratory of Cell Proliferation and Regulation Biology, Ministry of Education, Institute of Cell Biology, College of Life Sciences, Beijing Normal University, Beijing China; [2]State Key Laboratory of Pathogen and Biosecurity, Beijing Institute of Microbiology and Epidemiology, Beijing China

Fig. S1

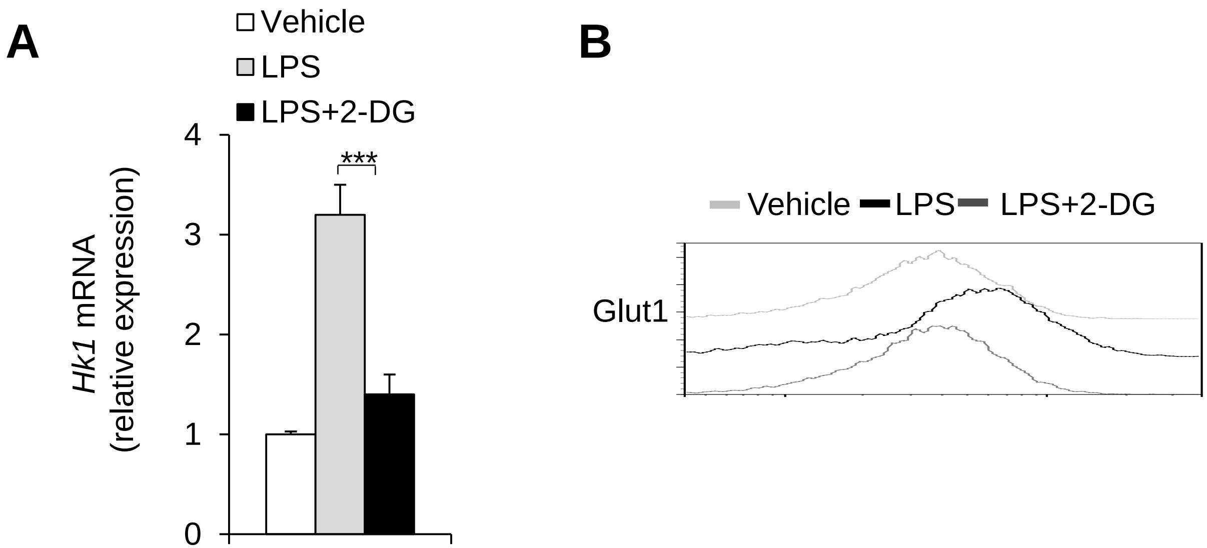

Fig. S1 Effects of 2-DG treatment on *Hk1* mRNA and glut 1 expressions.
PEMs sorted from C57BL/6 mice were stimulated with LPS (100 ng/mL) for 24 hrs, with or without glycolysis inhibitor, 2-DG. mRNA levels of *Hk1* were determined by qPCR, and glut 1 expressions were determined by flow cytometry assays. Data are representative of three individual experiments (n=3-5 mice per group). Statistical significance was measured by one-way ANOVA for comparisons among multiple groups. ***P <0.001, compared with the indicated groups.

Fig.S2

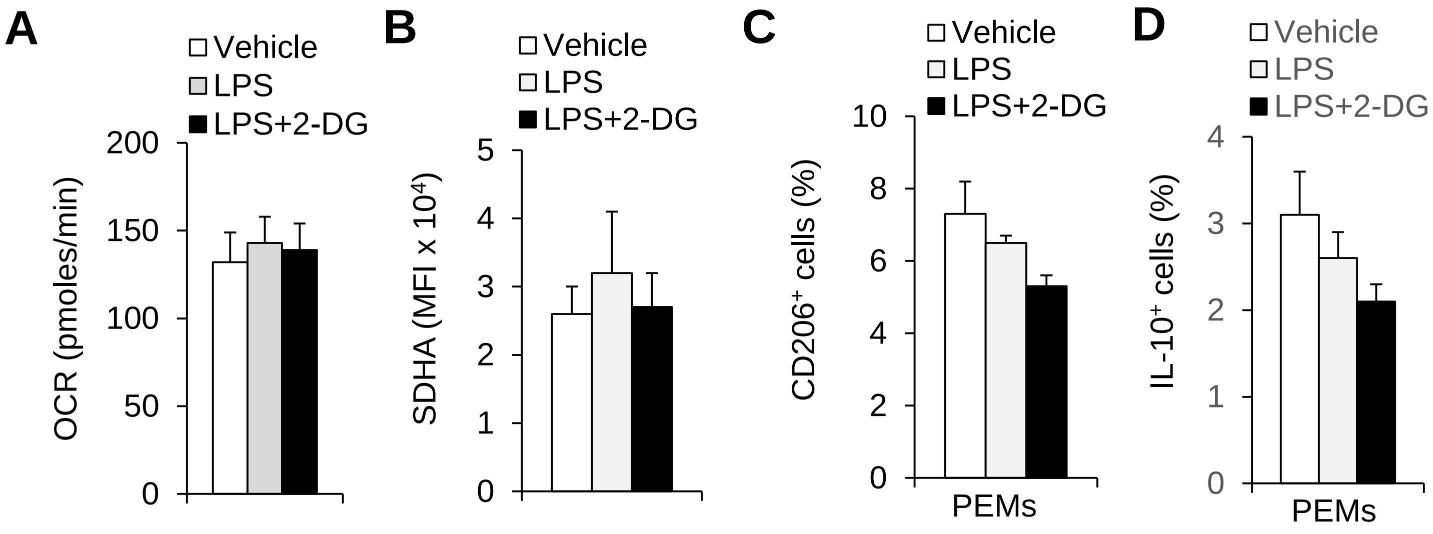

Fig. S2 2-DG treatment effects on PEMs.
PEMs sorted from C57BL/6 mice were stimulated with LPS (100 ng/mL) for 24 hrs, with or without glycolysis inhibitor, 2-DG (1 mM). (**A**) Cellular OXPHOS activity was measured by monitoring the OCR of cells. (**B**) Expressions of SDHA, CD206, and IL-10 were determined by flow cytometry. Data are representative of three individual experiments (n=3-5 mice per group).

Fig.S3

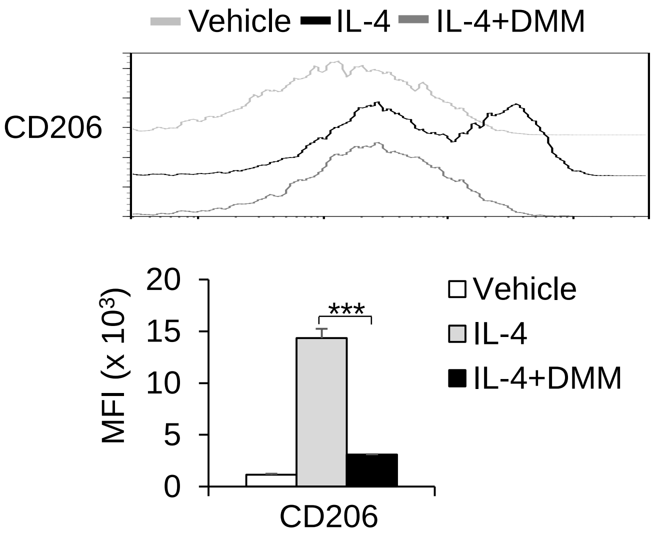

Fig. S3 OXPHOS is required for M2 macrophage polarization.
PEMs sorted from WT mice were pretreated with SDHA inhibitor DMM (160 mg/kg/mouse) and stimulated with IL-4 for 48 hrs. The protein levels of CD206 were determined by flow cytometry. Data are representative of three individual experiments (n=3-5 mice per group). Statistical significance was measured by one-way ANOVA for comparisons among multiple groups. ***P <0.001, compared with the indicated groups.

Fig.S4

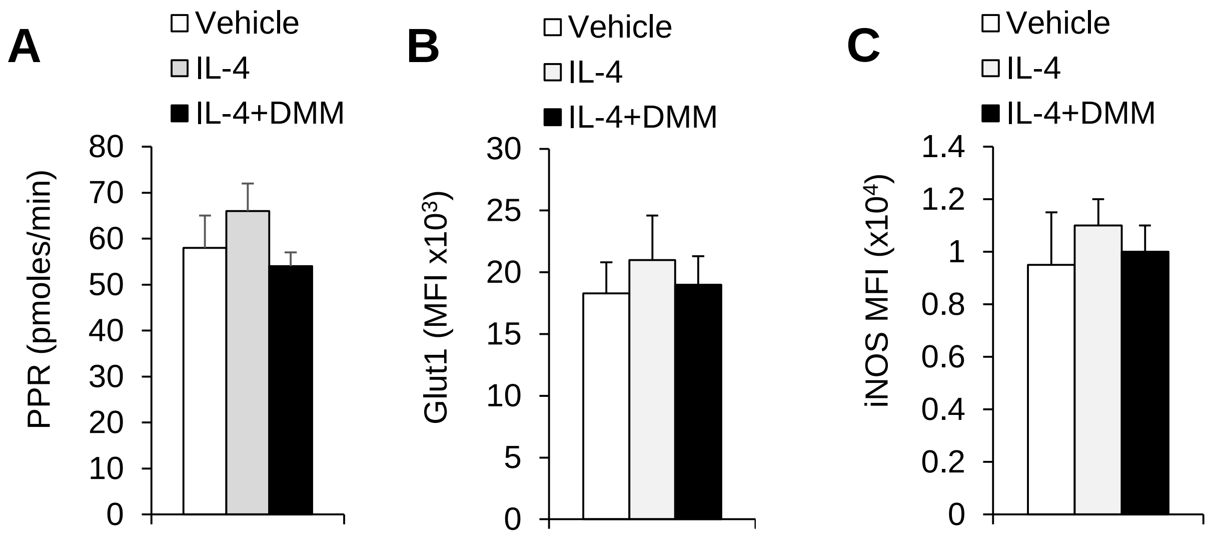

Fig. S4 DMM treatment effects on macrophages.
PEMs sorted from WT mice were pretreated with SDHA inhibitor DMM (160 mg/kg/mouse) and stimulated with IL-4 (10 ng/mL) for 48 hrs. The respiratory burst was determined by measuring the PPR (**A**). (**B-C**) Intracellular staining of Glut1 (**B**) and iNOS (**C**). Data are representative of three individual experiments (n=3-5 mice per group).

Fig.S5

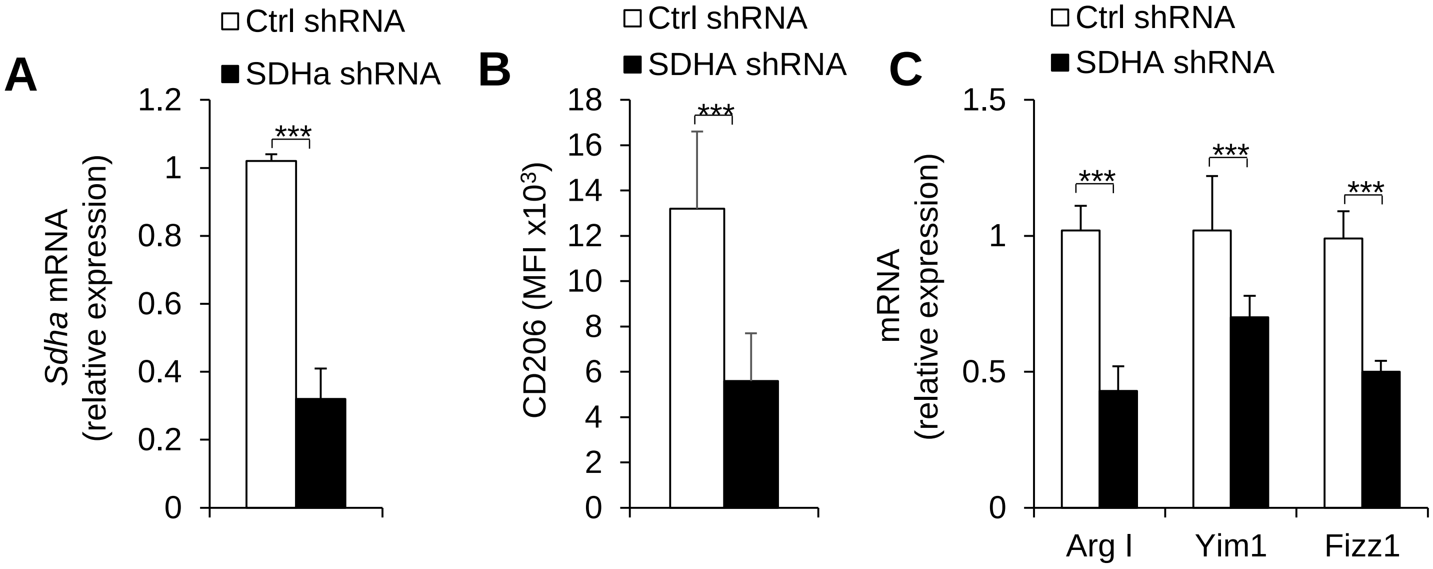

Fig. S5 Effects of *Sdha* shRNA in macrophages.

Indicated molecular expressions in the sorted BMDMs expressing control shRNA or SDHA shRNA vector (**A-C**). Data are representative of three individual experiments (n=3-5 mice per group). Student's unpaired *t* test for comparisons between two groups.****P*<0.001, compared with the indicated groups.

Fig.S6

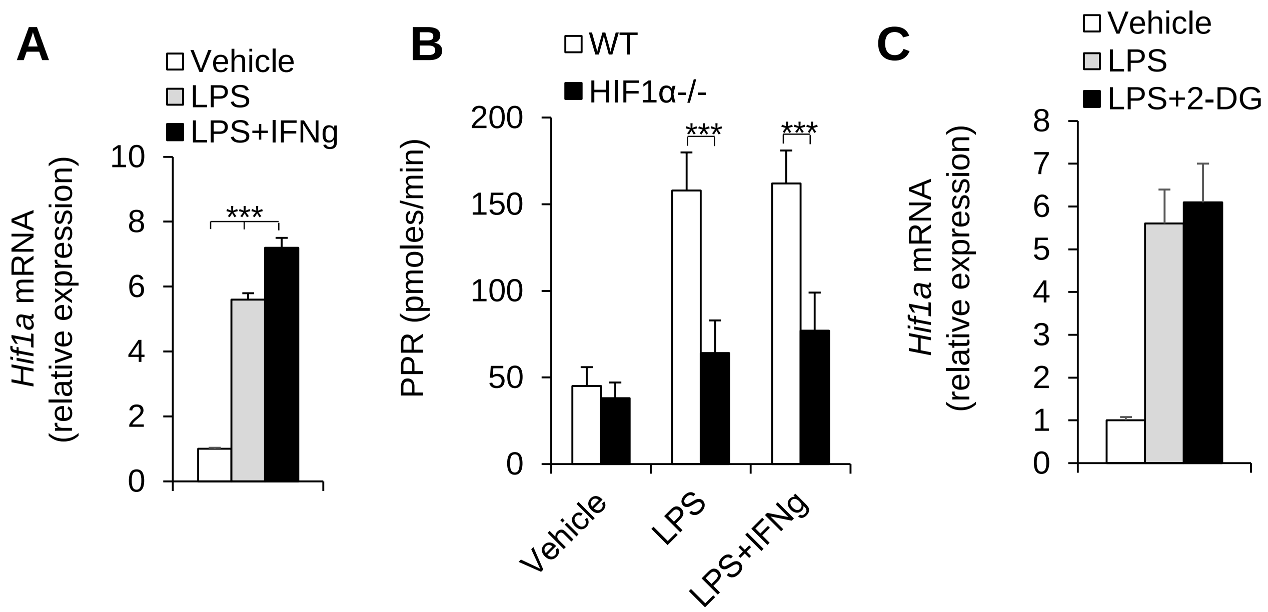

Fig. S6 HIF1α expressions under M1 macrophages differentiation conditions.
PEMs sorted from WT mice pretreated with glycolysis inhibitor 2-DG (1 mM) for 1 hr were stimulated with LPS (100 ng/mL) for 12 hrs. Indicated signal molecular were determined. Data are representative of three individual experiments (n=3-5 mice per group). Statistical significance was measured by one-way ANOVA for comparisons among multiple groups and Student’s unpaired *t* test for comparisons between two groups. ****P*<0.001, compared with the indicated groups.

Fig.S7

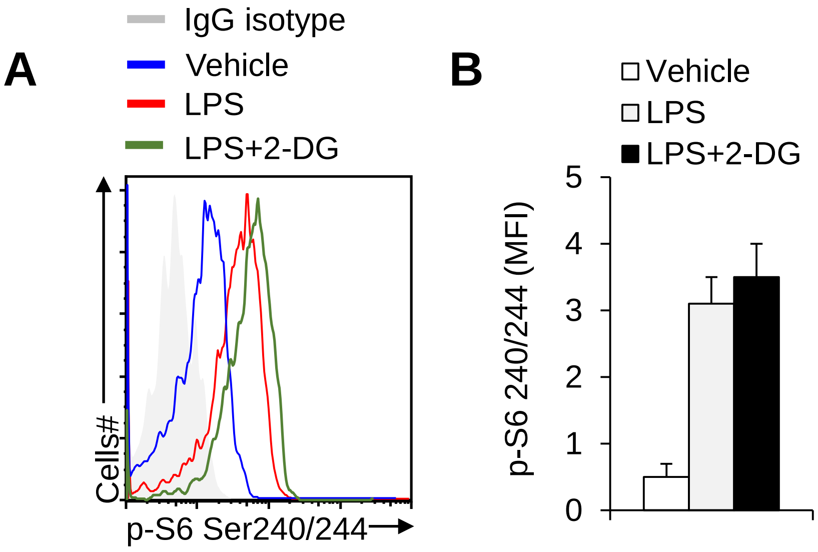

Fig. S7 Effects of 2-DG treatment on p-S6 expressions.
PEMs sorted from C57BL/6 mice were stimulated with LPS (100 ng/mL) for 24 hrs, with or without glycolysis inhibitor, 2-DG (1 mM). Protein expression of p-S6 Ser 240/244 were determined by flow cytometry assays. Data are representative of three individual experiments (n=3-5 mice per group).

Fig.S8

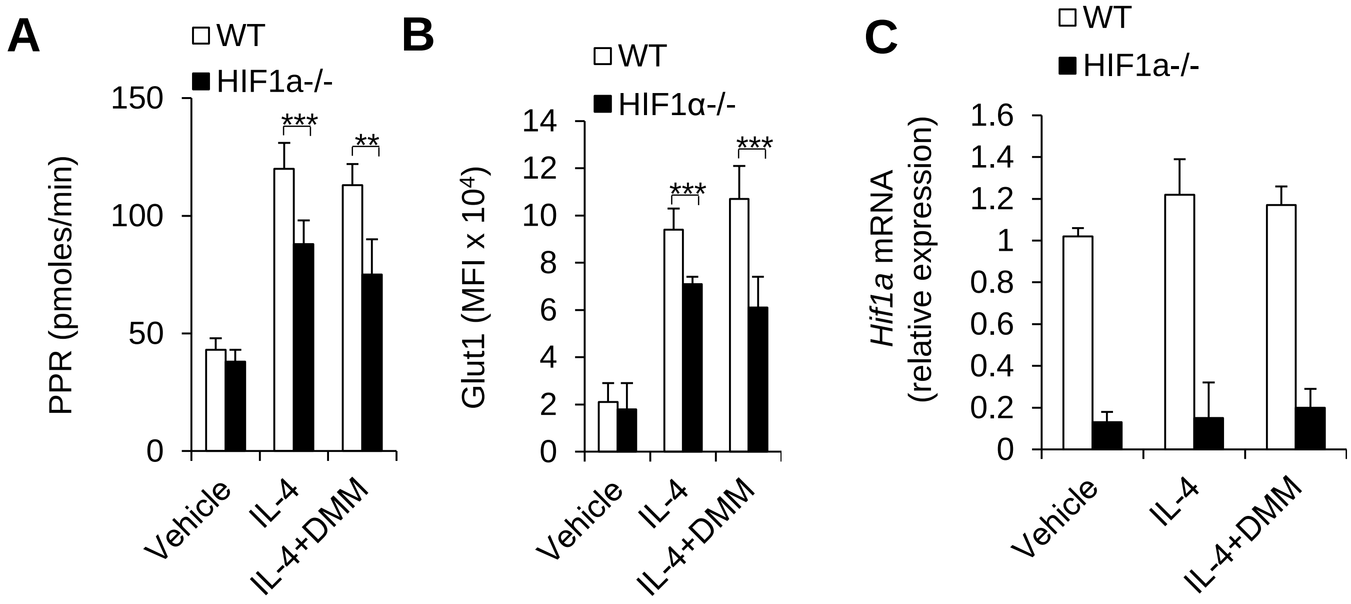

Fig. S8 HIF1α effects on M2 macrophages differentiations.
PEMs sorted from WT mice pretreated with DMM (10 mM) for 1 hr were stimulated with IL-4 (10 ng/mL) for 48 hrs. Indicated signal molecular were determined. Data are representative of three individual experiments (n=3-5 mice per group). Student's unpaired *t* test for comparisons between two groups.****P*<0.001, compared with the indicated groups.

Fig.S9

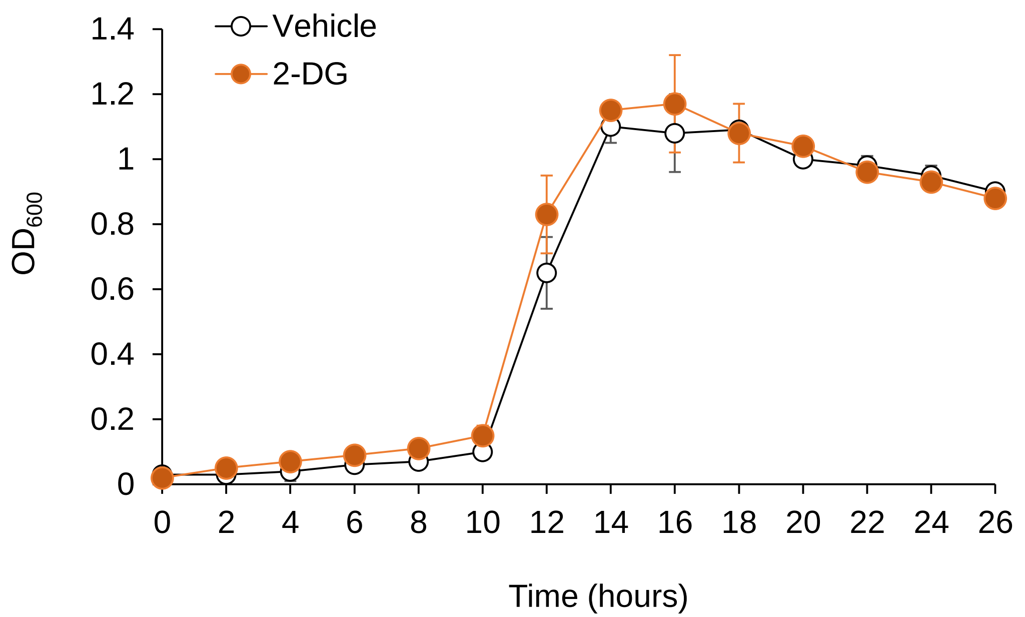

Fig. S9 Growth curve of *L. monocytogenes* bacteria.
L. Monocytogenes was cultivated in trypticase soy broth containing vehicle (PBS) or 2-DG (400 mM) and OD_{600} was detected as the indicated time with spectrophotometer and data analyzed. Data are representative of three individual experiments (n=6 per group)

Fig.S10

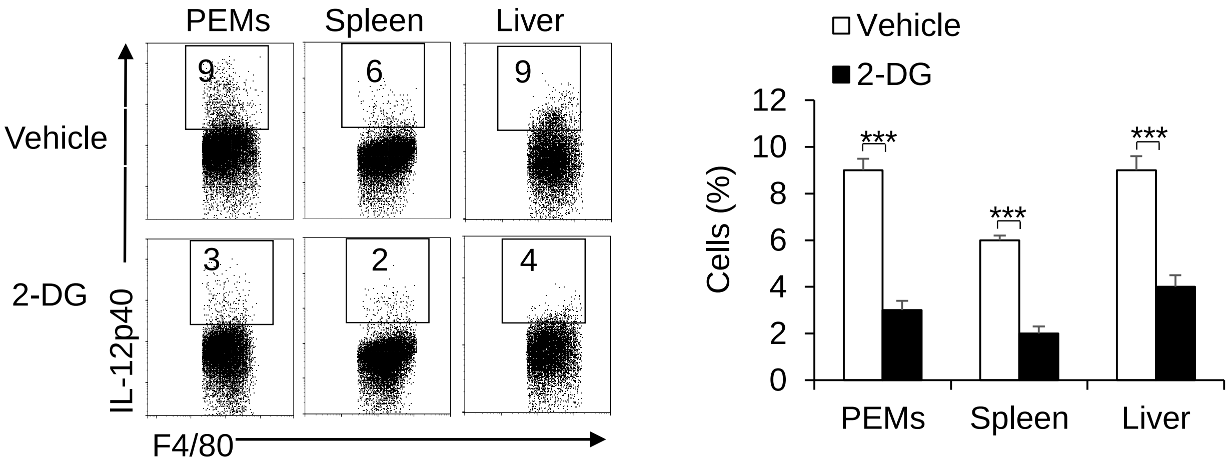

Fig. S10 Blocking glycolysis aggravates *L. monocytogenes* infections and suppresses the M1 macrophages differentiation. WT mice were injected i.p. with 2-DG (2 g/kg weight). 4 h later, mice were i.p. injected with 3 $\times$ 10^5 CFU of *L. monocytogenes* bacteria. 48 hrs after injection, mice were killed, and the CFU of mouse livers, spleens and peritoneal cavity were determined. Macrophages from peritoneal cavity, spleen and livers were restimulated with LPS (100 ng/mL) for 5 hrs, together with GolgiStop, and the protein expressions of pro-inflammatory cytokines IL-12p40 were determined by flow cytometry assays. Data are representative of three individual experiments (n=3-5 mice per group). Student's unpaired *t* test for comparisons between two groups.****P*<0.001, compared with the indicated groups.

Fig.S11

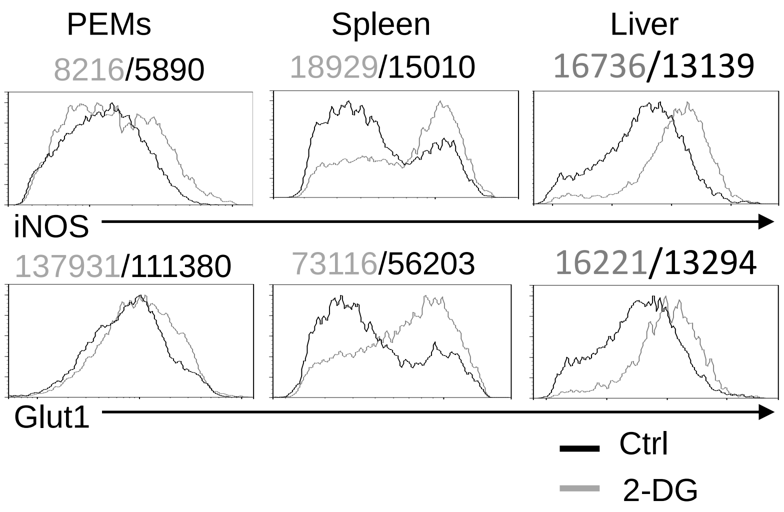

Fig. S11 Expressions of iNOS and Glut1 in macrophages from *L. monocytogenes* infected mice.
WT mice were injected i.p. with 2-DG (2 g/kg weight). 4 hr later, mice were i.p. injected with 3×10^5 CFU of *L. monocytogenes* bacteria. 48 hrs after injection, mice were killed. Macrophages from peritoneal cavity (PEMs), spleen and livers were re-stimulated with LPS (100 ng/mL) for 5 hrs, together with GolgiStop, and the protein expressions of iNOS and glut1 were determined by flow cytometry. Data are representative of three individual experiments (n=3-5 mice per group).

Fig.S12

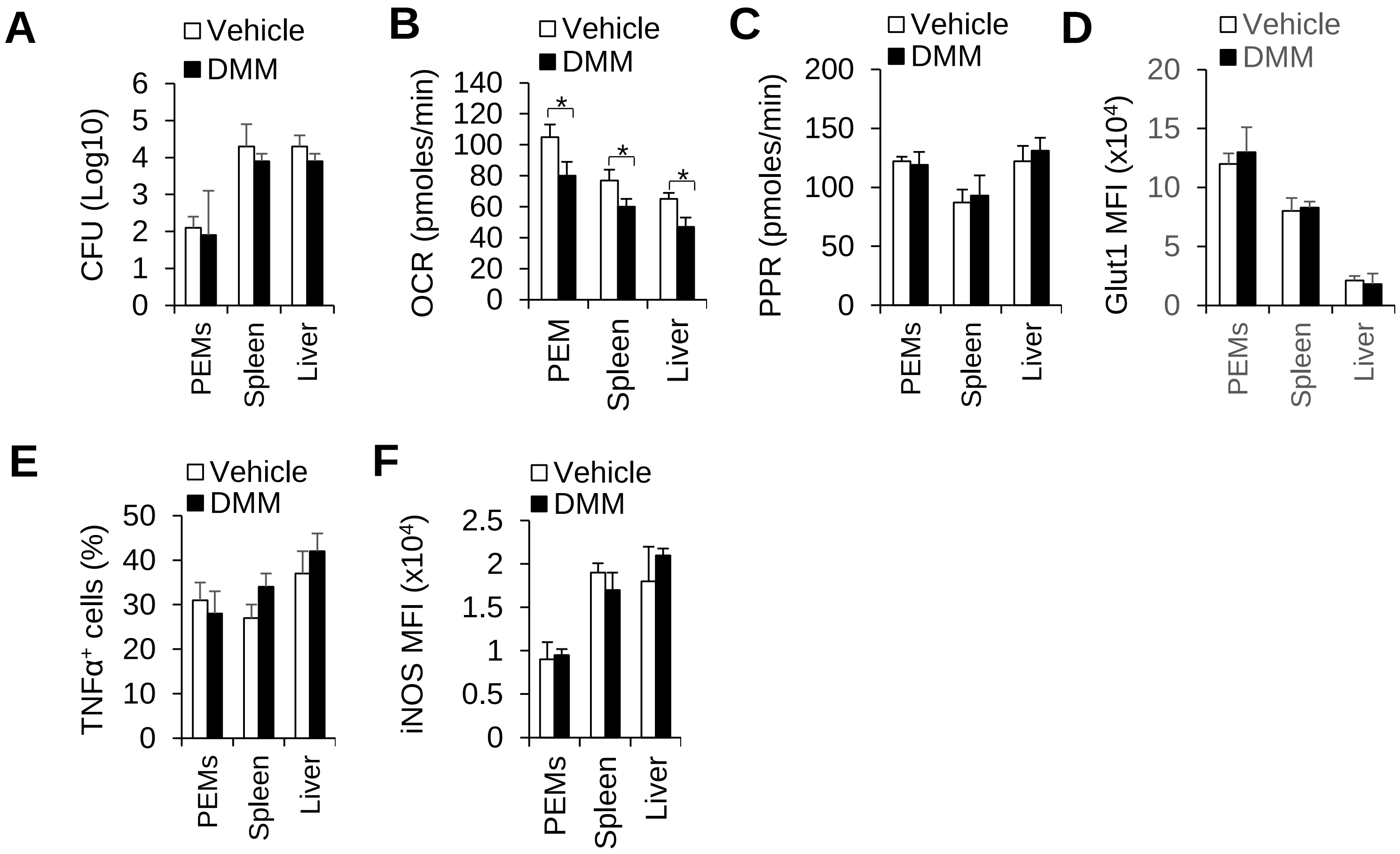

Fig. S12 Effects of DMM treatment on macrophages during the *L. monocytogenes* infections.
WT mice were injected i.p. with DMM (160 mg/kg/mouse). 4 hr later, mice were i.p. injected with 3×10^5 CFU of *L. monocytogenes* bacteria. 48 hrs after injection and indicated markers of macrophages were determined. Data are representative of three individual experiments (n=3 mice per group). Student's unpaired *t* test for comparisons between two groups.**P*<0.05, compared with the indicated groups.

Fig.S13

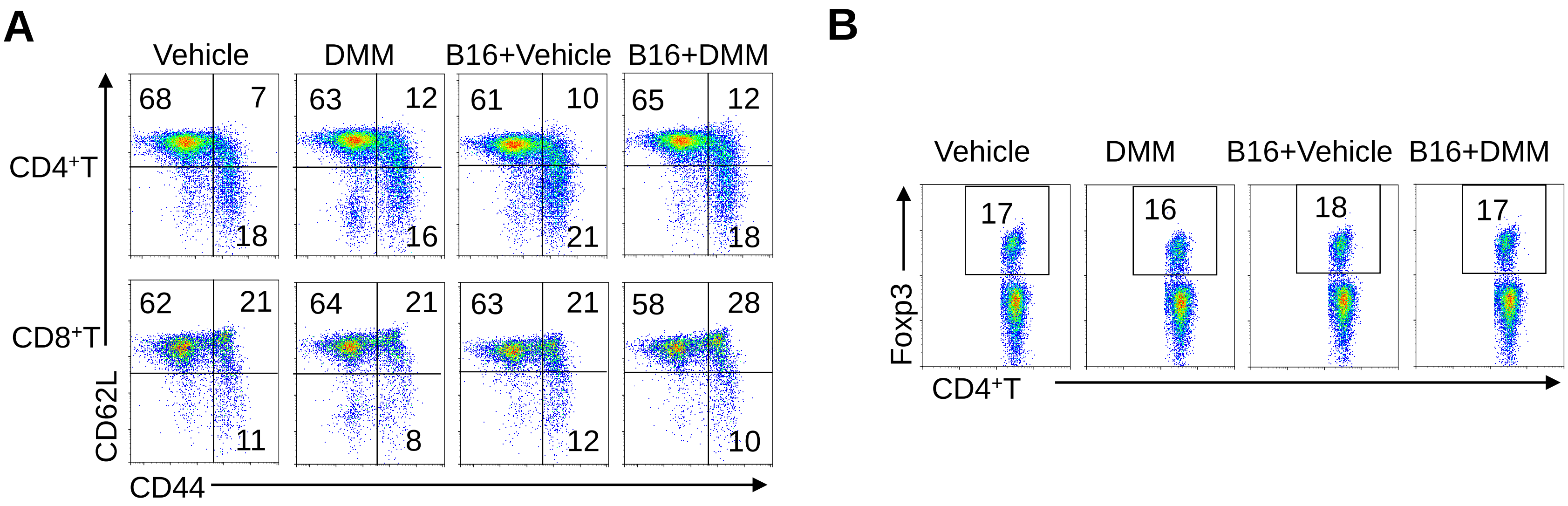

Fig. S13 DMM effects on T cells in anti-tumor immunity.

WT mice were i.p. injected with DMM (160 mg/kg/mouse) for 4 hrs and s.c. injected with 2 x 10^5 B16F10 melanoma cells. The percentages of effector T cells (**A**) and T_{reg} cells (**B**) from the draining lymph nodes (dLN) were determined by flow cytometry. Data are representative of three individual experiments (n=3-5 mice per group).

Fig.S14

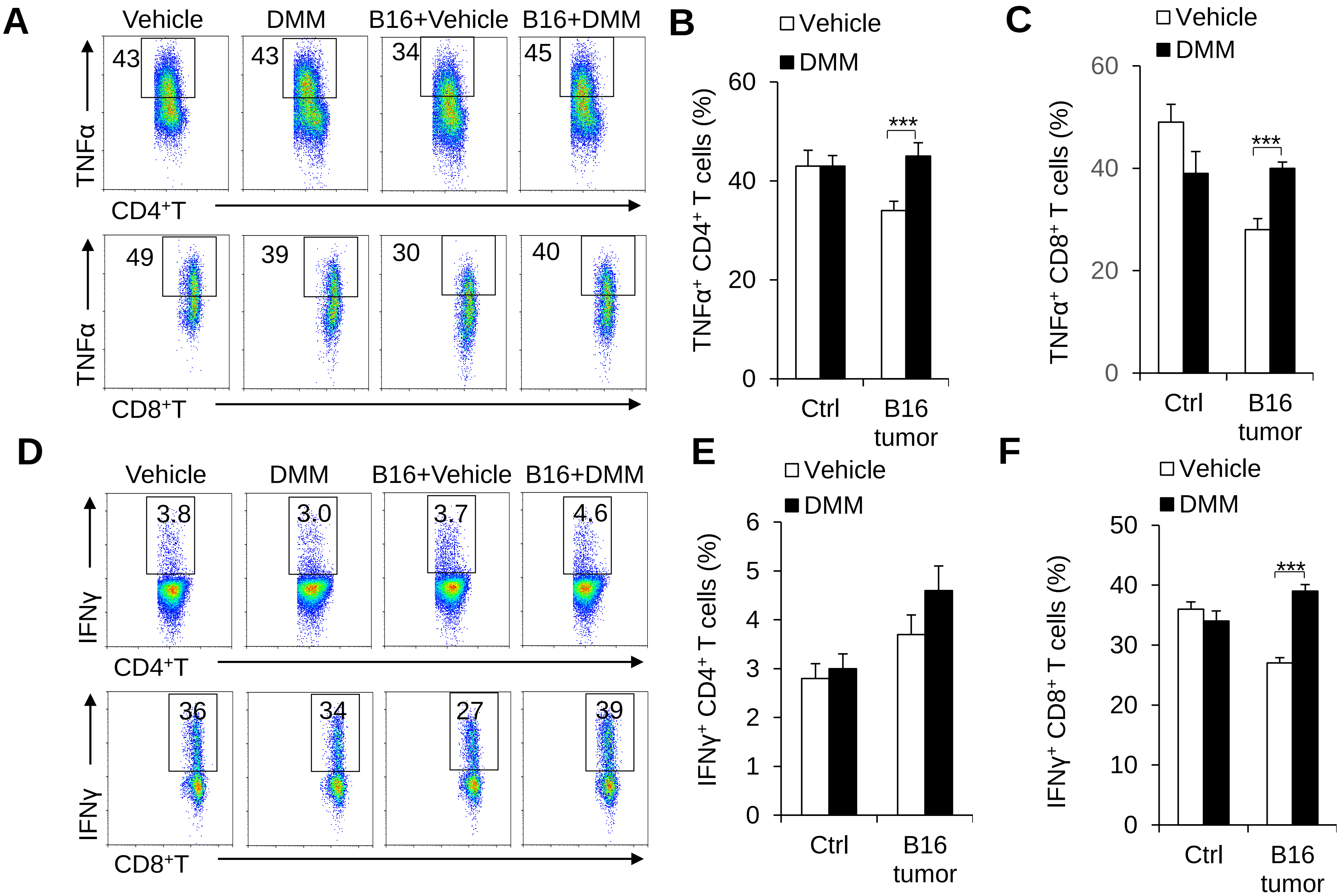

Fig. S14 Role of OXPHOS signal pathway in anti-tumor immunity.
C57BL/6 mice were i.p. injected with DMM (160 mg/kg/mouse) for 4 hrs and s.c. injected with 2X10^5 B16F10 melanoma cells. TNFα (**A-C**) and IFNγ (**D-E**) production by $CD4^+$ or $CD8^+$ T cells were determined by flow cytometry assays. Data are representative of three individual experiments (n=3-5 mice per group). Student's unpaired *t* test for comparisons between two groups.***P<0.001, compared with the indicated groups.

Fig.S15

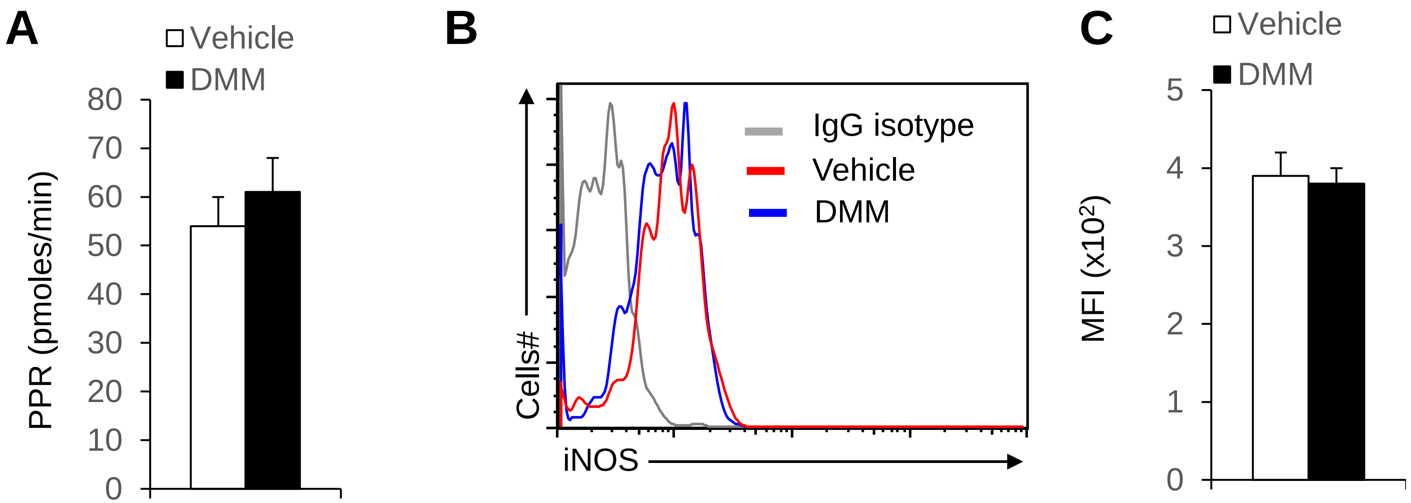

Fig. S15 DMM effects on T cells in anti-tumor immunity.

WT mice were i.p. injected with DMM (160 mg/kg/mouse) for 4 hrs and s.c. injected with 2 x 10^5 B16F10 melanoma cells. Macrophages isolated from tumor and the respiratory burst was determined by measuring the PPR (**A**) and intracellular staining of iNOS (**B-C**) in macrophages were analyzed with flow cytometry. Data are representative of three individual experiments (n=3-5 mice per group).

Fig.S16

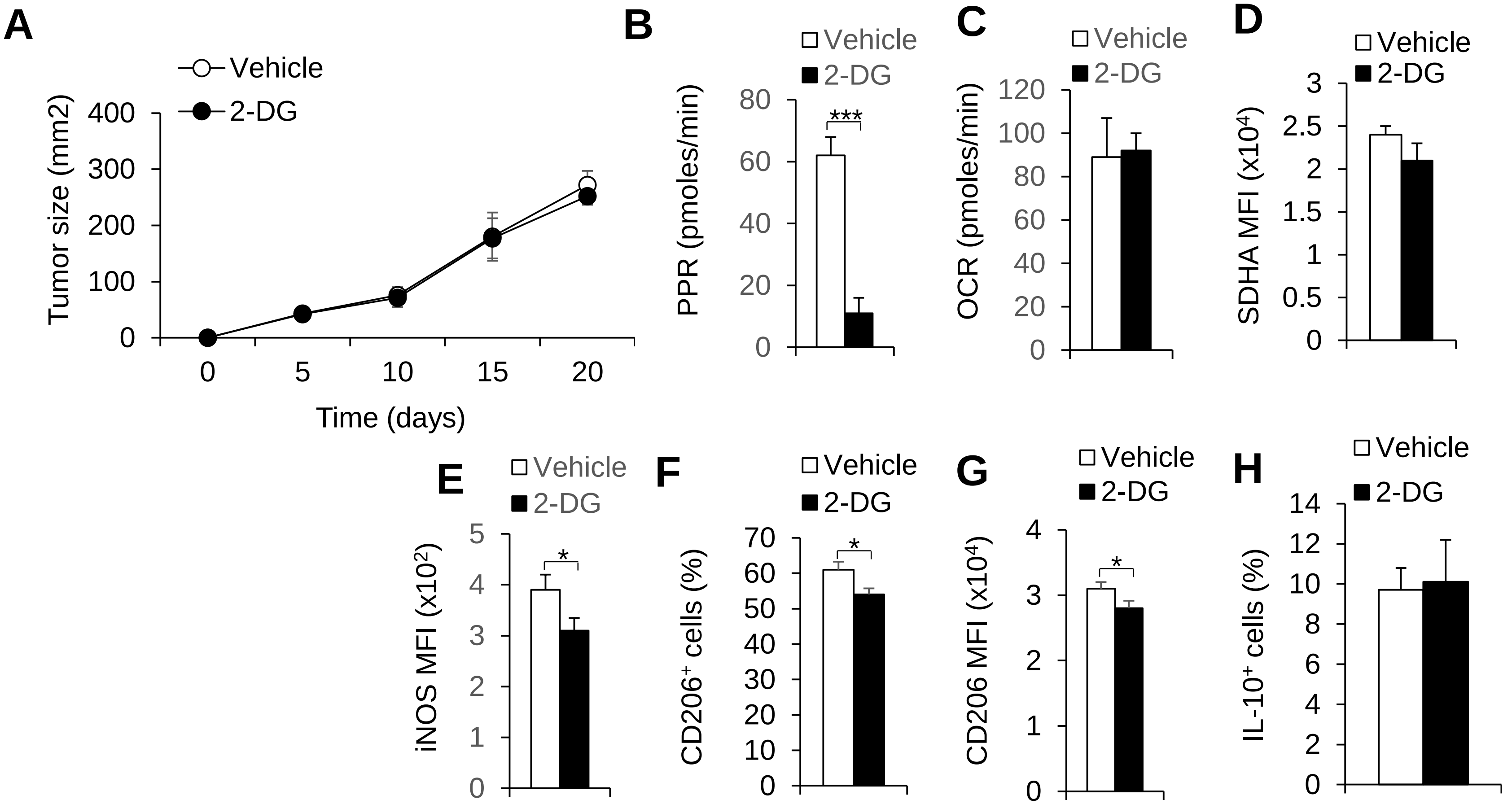

Fig. S16 2-DG effects on macrophages in anti-tumor immunity.

WT mice were i.p. injected with 2-DG (2 g/kg weight) for 4 hrs and s.c. injected with 2 x 10^5 B16F10 melanoma cells.(**A**) Tumor growth curve. (**B-H**) Macrophages isolated from tumor and the respiratory burst was determined by measuring the PPR (**B**) and cellular OXPHOS activity was measured by monitoring the OCR of cells (**C**). intracellular staining of SDHA and iNOS (**D-E**) in macrophages were analyzed with flow cytometry. CD206 percent and MFI in macrophages in tumor (**F-G**). Intracellular staining of IL-10 in macrophages in tumor. Data are representative of three individual experiments (n=3-5 mice per group). Student's unpaired *t* test for comparisons between two groups. **P*<0.05 and ****P* <0.001, compared with the indicated groups.

Fig.S17

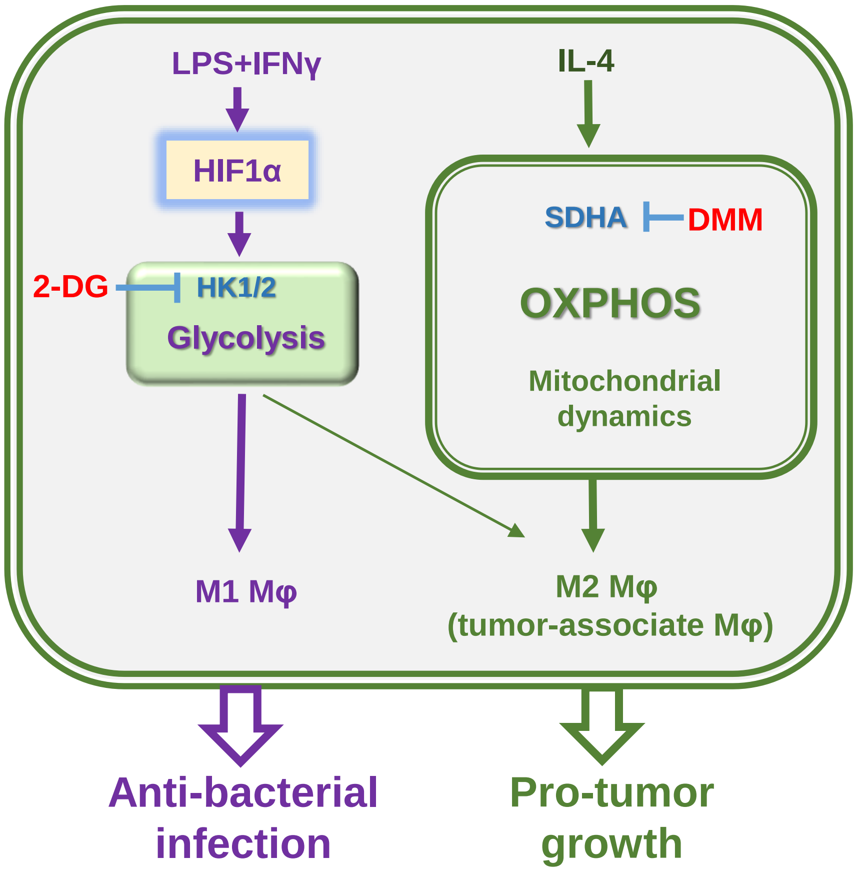

Fig. S17 Regulations of glycolytic activities on macrophages functions in infection and cancer. Proposed model how glycolysis and OXPHOS in macrophages (Mφ) integrate the innate stimuli to regulate the function and programming of macrophage subpopulation in infectious and cancerous environment through metabolic dependent mechanisms.
